# Azole sensitivity in *Leptosphaeria* pathogens of oilseed rape: the role of lanosterol 14α-demethylase

**DOI:** 10.1038/s41598-017-15545-9

**Published:** 2017-11-20

**Authors:** Thomas R. Sewell, Nichola J. Hawkins, Henrik U. Stotz, YongJu Huang, Steven L. Kelly, Diane E. Kelly, Bart Fraaije, Bruce D. L. Fitt

**Affiliations:** 1 Centre for Agriculture, Food and Environmental Management, University of Hertfordshire, Hatfield, UK; 2 Department of Infectious Disease Epidemiology, Imperial College London, London, UK; 3 Biointeractions and Crop Protection, Rothamsted Research, Harpenden, Hertfordshire, UK; 4 Centre for Cytochrome P450 Biodiversity, Institute of Life Science, School of Medicine, Swansea University, Swansea, UK

## Abstract

Lanosterol 14-α demethylase is a key enzyme intermediating the biosynthesis of ergosterol in fungi, and the target of azole fungicides. Studies have suggested that *Leptosphaeria maculans* and *L. biglobosa*, the causal agents of phoma stem canker on oilseed rape, differ in their sensitivity to some azoles, which could be driving pathogen frequency change in crops. Here we used CYP51 protein modelling and heterologous expression to determine whether there are interspecific differences at the target-site level. Moreover, we provide an example of intrinsic sensitivity differences exhibited by both *Leptosphaeria* spp. *in vitro* and *in planta*. Comparison of homologous protein models identified highly conserved residues, particularly at the azole binding site, and heterologous expression of LmCYP51B and LbCYP51B, with fungicide sensitivity testing of the transformants, suggests that both proteins are similarly sensitive to azole fungicides flusilazole, prothioconazole-desthio and tebuconazole. Fungicide sensitivity testing on isolates shows that they sometimes have a minor difference in sensitivity *in vitro* and *in planta*. These results suggest that azole fungicides remain a useful component of integrated phoma stem canker control in the UK due to their effectiveness on both *Leptosphaeria* spp. Other factors, such as varietal resistance or climate, may be driving observed frequency changes between species.

## Introduction

Antifungal demethylation inhibitors (DMIs), e.g. triazoles and imidazoles (azoles), target lanosterol 14α-demethylase (CYP51, *erg11*), a member of the cytochrome P450 superfamily and key regulatory enzyme in the ergosterol biosynthetic pathway^1,2^. Ergosterol is an essential structural component of the fungal plasma membrane and is required to maintain membrane integrity, fluidity and permeability. Inhibition of CYP51 blocks the synthesis of ergosterol, which subsequently increases cytotoxic sterol precursors. The lack of membrane-bound ergosterol and the accumulation of toxic metabolites in the cytosol have a combined fungistatic effect on the cell, decreasing membrane integrity and permeability^3^.

Azoles are used to control fungal pathogens in both clinical and agricultural situations. In clinical settings, voriconazole, itraconazole and posaconazole are commonly used to treat the opportunistic human pathogen *Aspergillus fumigatus*, which can cause invasive pulmonary infections amongst immunocompromised patients^4^, along with other invasive pathogens, e.g. *Candida* spp^5^. In agriculture, a wide range of azole fungicides have been used to control fungal plant pathogens since their inception in the 1970s^6^. Since 2005, azole fungicides have maintained approximately 20% share of the global fungicide market, which in total (including seed treatments) was worth $13.3 billion in 2011^6–8^.

Agricultural azole fungicides are used as a component of fungicide programmes (often in mixtures) to control fungal diseases, including phoma stem canker of winter oilseed rape (*Brassica napus*) caused by coexisting fungal plant pathogens *Leptosphaeria maculans* and *L. biglobosa* ^9^. Globally, phoma stem canker causes approximately £700 million worth of yield losses, making it a considerable threat to sustainable oilseed rape production and food security^10^.

Recent studies have suggested that the two *Leptosphaeria* spp. differ in their sensitivity to azoles, both *in vitro* ^11^ and *in planta* ^12^, with *L. biglobosa* exhibiting a less sensitive phenotype in the presence of flusilazole and tebuconazole. Differences in sensitivity between coexisting plant pathogens can affect population structure^13^ and it has been suggested that lower azole sensitivity could explain the recent increase in *L. biglobosa* incidence in some UK locations^14^.

Increased exposure to azole fungicides has led to the evolution of resistance in some plant pathogen populations. Currently, four mechanisms of resistance to DMIs have been identified in agricultural crop pathogens; these are (1) target site polymorphisms (2) CYP51 overexpression (3) presence of CYP51 paralogs (CYP51A, CYP51B, CYP51C) and (4) increased expression of efflux pump genes^6,15,16^. Missense mutations in the coding sequence of *erg11*, the gene that encodes CYP51, and subsequent alterations in the secondary structure of CYP51, correlate with an insensitivity to DMI fungicides and are currently the most common mechanism of azole-resistance^6,15,17–19^. Using predictive homology modelling, polymorphisms in the CYP51 coding sequence have shown to directly interfere with binding and/or increase the size of the azole binding pocket, thus decreasing the affinity of the bound fungicide and reducing its effectiveness as an anti-fungal^20^.

Using predictive homology modelling supported by heterologous expression in a *Saccharomyces cerevisiae* mutant, we have investigated if CYP51 structural differences play a role in fungicide-sensitivity differences previously reported between *Leptosphaeria maculans* and *L. biglobosa* populations. Moreover, using a subset of isolates from south east UK, we sought to confirm established reports of fungicide sensitivity in *L*. *maculans and L*. *biglobosa* and provide a mechanistic example of this both *in vitro* and *in planta*.

## Results

### Characterisation and sequencing of Lm*erg11*/CYP51B and Lb*erg11*/CYP51B

All 12 *L. maculans* isolate sequence assemblies (GenBank accession numbers KY500978 – KY500989) encompassed Lm*erg11*, which was 1635 bp in length and consisted of two exons and one 54 bp intron; nucleotide sequence similarity between all isolates was 100%. LmCYP51B was identical in all 12 isolates but differed by 1 amino acid (K337N) when compared with the published LmCYP51B sequence (AAN28927.1) from an Australian isolate (M1). Lb*erg11* was conserved within all *L. biglobosa* sequence assemblies (GenBank accession numbers KY500970–KY500977), was 1642 bp in length and consisted of two exons and one 58 bp intron. Of the eight isolates sequenced, six had an identical *erg11* gene, while two isolates (F2 dm 11-5 and H Dr 12 12) contained a synonymous single nucleotide polymorphism (SNP), namely C1467T; therefore all 8 protein sequences were 100% similar. Neither *Leptosphaeria* spp. contained amino acid alterations previously recognised as conferring reduced azole sensitivity in other plant pathogenic fungi^6^.

A pair-wise alignment between LmCYP51B and LbCYP51B identified 24 amino acid alterations, one amino acid deletion and a pair-wise percentage identity of 97.5% (Score matrix = BLOSUM80, threshold = 1) (SF 2). Of the 24 alterations, 12 were considered a change to amino acids with similar physical-chemical properties and 12 considered a change to amino acids with dissimilar physical-chemical properties (Score matrix = BLOSUM80, threshold = 1).

**Figure 1:**
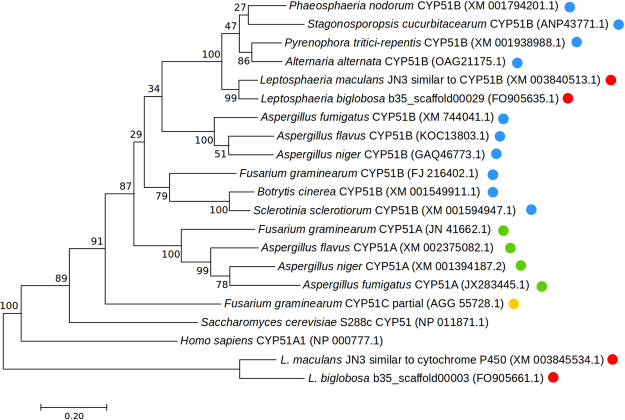
Maximum Likelihood phylogenetic tree of fungal CYP51 sequences including all three CYP51 paralogs: CYP51A (green) CYP51B (blue) and CYP51C (yellow). Closest match to CYP51A and CYP51C in *Leptosphaeria* spp. genomes are also displayed, as are the *Leptosphaeria* spp. CYP51B paralogs identified in this study (red). The evolutionary history was inferred by using the maximum likelihood method based on the general time reversible model^41^. Branch lengths scaled by number of substitutions per site. Node labels indicate percentage bootstrap support (1000 replicates).

Phylogenetic analysis by maximum likelihood clearly shows that CYP51B from *L. maculans* and *L. biglobosa* are sister lineages within the *Pezizomycotina* CYP51B clade (Fig. 1). CYP51B is a CYP51 paralog found in all filamentous ascomycetes. An additional paralog and functioning sterol 14-α demethylase, CYP51A, for which high levels of expression are associated with reduced azole sensitivity in some filamentous ascomycetes^16^, was not found in either *Leptosphaeria* species. Moreover, CYP51C, a non-functionalised paralog found in *Fusarium* spp.^21^, was not identified in either of the *Leptosphaeria* spp. genomes (Fig. 1).

### Predictive homology modelling

**Figure 2:**
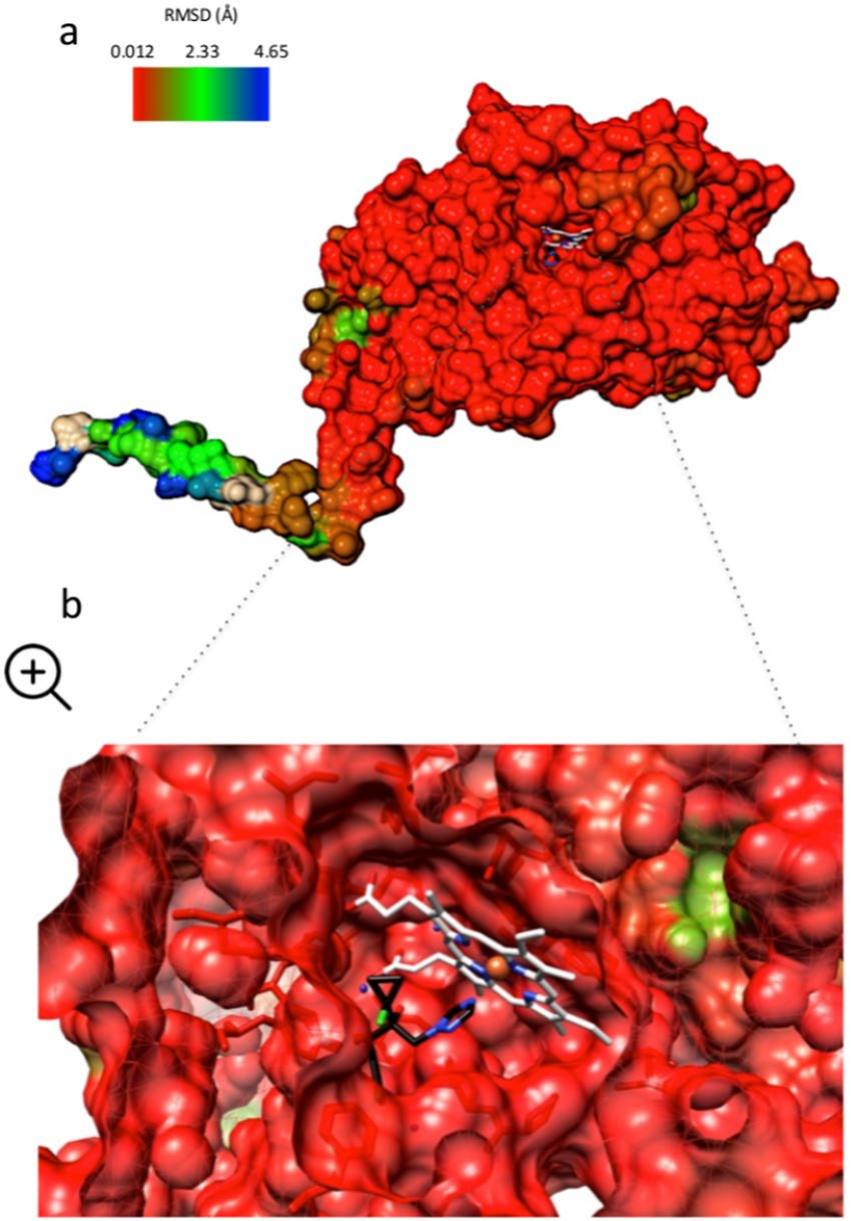
Structural pair-wise alignment (Match >Align; Chimera) on the predicted homology models of LmCYP51B and LbCYP51B (**a**). Difference in colour represents distance (Å) between aligned residues (RMSD), with red representing residues close in structural locality and blue representing residues that are further apart. Zoomed image (**b**) is a cross-section of the binding pocket of aligned proteins. Heme cofactor is coloured in white and bound azole ligand in black. Images were generated using Chimera.

**Figure 3:**
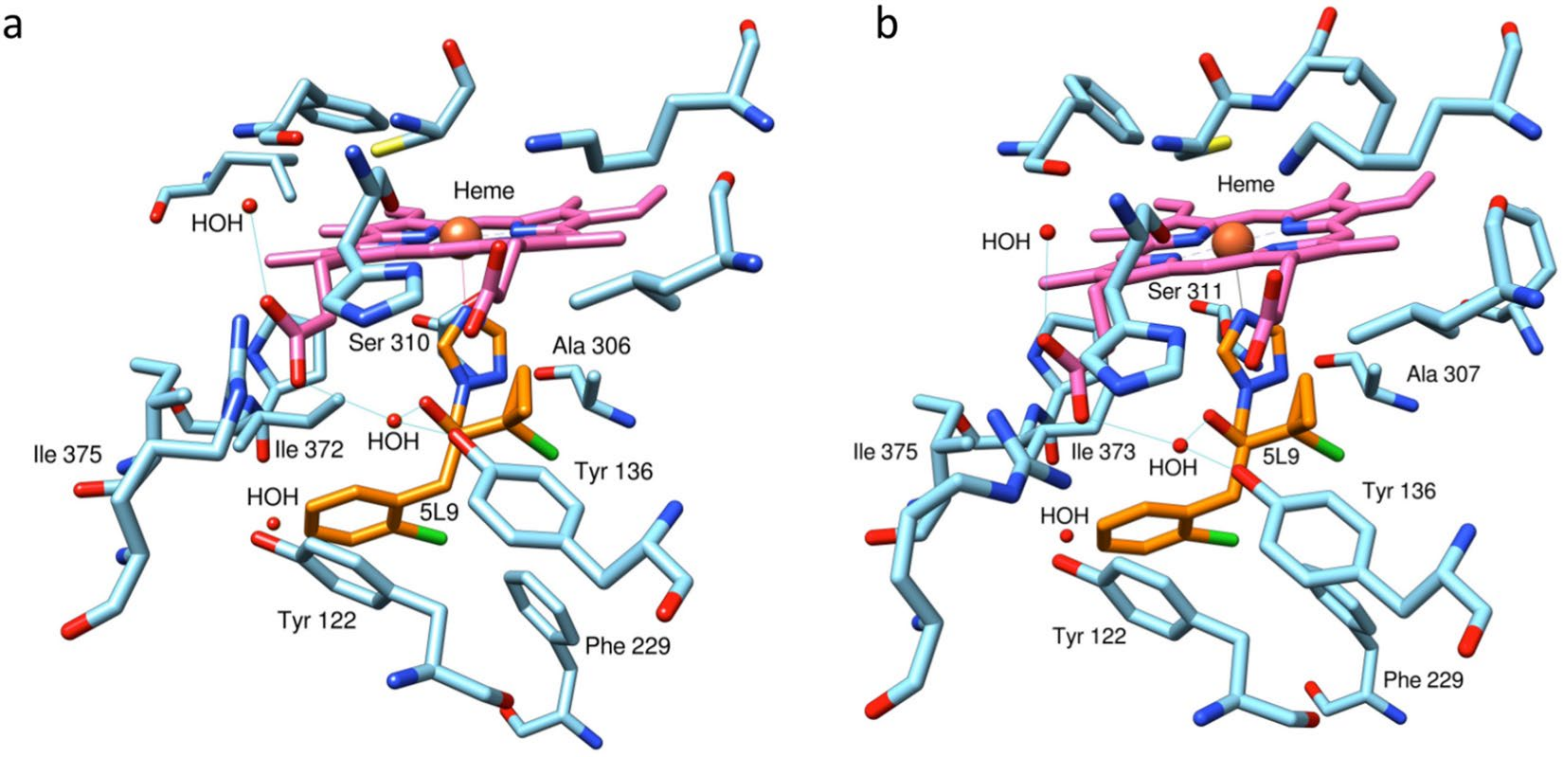
Azole binding sites in the *Leptosphaeria maculans CYP51B* (**a**) and *L. biglobosa CYP51B* (**b**) proteins inferred by homology. Superimposed prothioconazole-desthio (5L9) is highlighted in orange with residues <5 Å from ligand (5L9) labelled. The heme cofactor is highlighted in pink and the metal-ligand bond in purple. Hydrogen bonds are highlighted in blue and water molecules (HOH) in red. Images were generated in Chimera.

For both species, all five CYP51B models that were predicted by Modeller had a very high structural fold confidence (P < 0.001) and a global model quality score of 0.89 (0 to 1 scale). The best models for each species, according to ModFOLD4, were selected for further analysis. The predicted structures of LmCYP51B and LbCYP51B were very similar (TM-score = 0.90). The root-mean-square deviation (RMSD) of the common residues was 2.76 Å on a 526 amino acid alignment length (Fig. 2). The globular active sites of the proteins appear to have the highest structural conservation. Noteworthy differences in the predicted structures were distinguishable only in the trans-membrane tail, although some amino acid substitutions did slightly alter the predicted fold of the globular structure. The heme cofactor and subsequently bound prothioconazole-desthio ligand were shown to be located inside the binding pocket of the proteins. The surrounding amino acids, which form this pocket, were all highly conserved (Fig. 3).

Detailed two-dimensional schematics of protein-ligand interactions identified further similarities between the predicted structures of LmCYP51B (SF 3a) and LbCYP51B (SF 3b). A water-mediated hydrogen bond network was maintained in both structures, specifically facilitated by the presence of Tyr136, a water molecule and the relative location of the heme cofactor. Furthermore, five other residues, Tyr122, Ala306 (Ala307 in LbCYP51B), Ile372 (Ile373 in LbCYP51B), Ser374 (Ser375 in LbCYP51B) and Phe508 (Phe509 in LbCYP51B), which were interacting with the bound ligand (prothioconazole-desthio), were correspondingly located in the binding pocket (Fig. 3). The predicted structure of LbCYP51B had two extra residues, Ser311 and Leu508, both located sufficiently close to interact with the ligand.

### Heterologous expression of LmCYP51B and LbCYP51B in *Saccharomyces cerevisiae*

**Figure 4:**
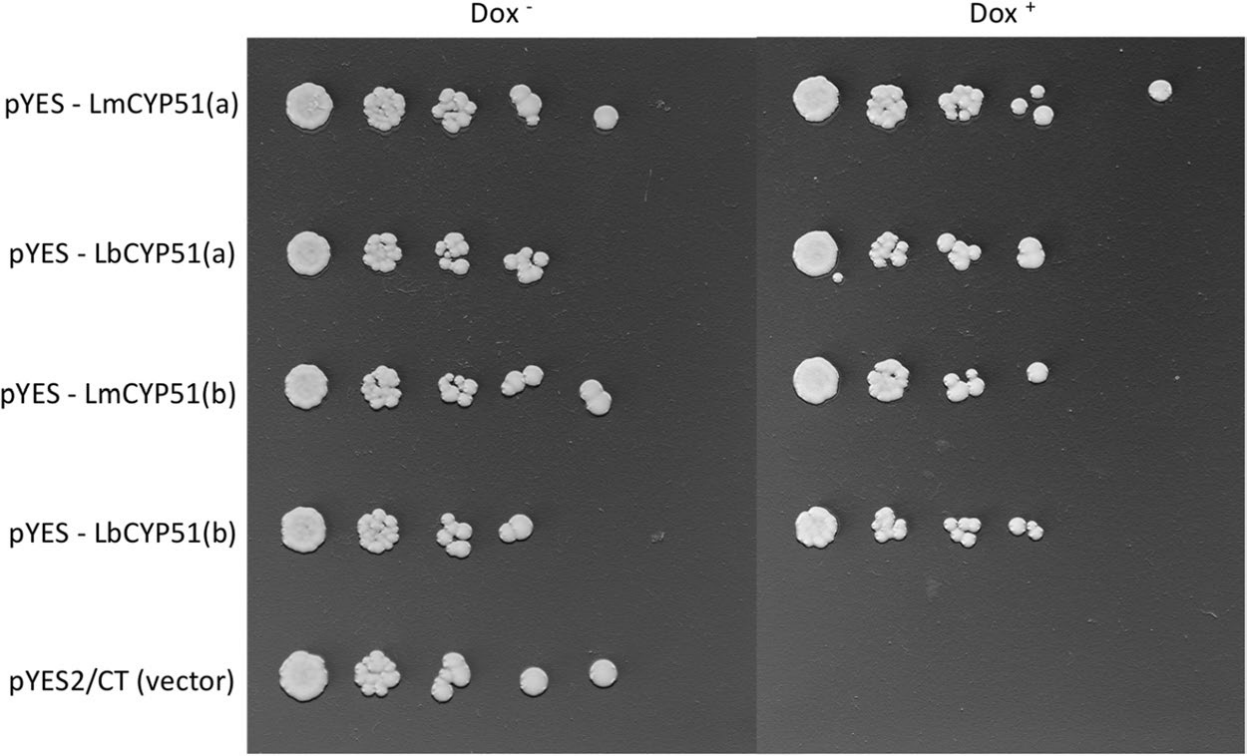
Complementation of *Saccharomyces cerevisiae* strain *YUG37::erg11* with *Leptosphaeria maculans* CYP51B and *Leptosphaeria biglobosa* CYP51B. Yeast transformants grown for 16 hours in SD + GAL + RAF media were inoculated onto SD + GAL + RAF agar without (DOX^−^) or with (DOX^+^) doxycycline, which represses the native *S. cerevisiae* CYP51B. The YUG37::pYES2/CT vector-only strain was used as a control. Lettering (**a,b**) refers to biological replicates.

The vector-only transformed strain YUG37::pYES2/CT, which lacked a complementary CYP51B protein, was unable to grow on the doxycycline amended SD GAL + RAF medium due to the repression of native CYP51B expression in *S. cerevisiae*. Complementation of native CYP51B was achieved in YUG37::LmCYP51B and YUG37::LbCYP51B *S. cerevisiae* transformants, which both showed robust growth across five 5-fold dilutions on SD GAL + RAF medium amended with doxycycline; demonstrating that expression of the predicted wild-type CYP51B protein from *L. maculans* or *L. biglobosa* can support ergosterol synthesis in *S. cerevisiae* (Fig. 4).

All YUG37 transformants expressing LmCYP51B or LbCYP51B were very sensitive to tebuconazole, flusilazole and prothioconazole-desthio (Table 1). There was a marginal, 1.35-fold (1 df, *P* < 0.05) difference between the EC_50_ values of YUG37::LmCYP51B and YUG37::LbCYP51B transformants for tebuconazole, and no significant difference in sensitivity to flusilazole or prothioconazole-desthio.

**Table 1:** Fungicide sensitivity (EC_50_ µg ml^−1^) of YUG37::LmCYP51B and YUG37::LbCYP51B *Saccharomyces cerevisiae* transformants. Standard deviations (±) were calculated from two biological replicates.

Transformant	Tebuconazole	Fold difference	Flusilazole	Fold difference	Prothioconazole-desthio	Fold difference
YUG37::LmCYP51B	0.0084 (0.0017)		0.036 (0.014)		0.0007 (0.0006)	
YUG37::LbCYP51B	0.0062 (0.0005)	1.35*	0.032 (0.0056)	**1.13**	0.0004 (0.0002)	**1.75**

### Fungicide sensitivity *in vitro*

**Figure 5:**
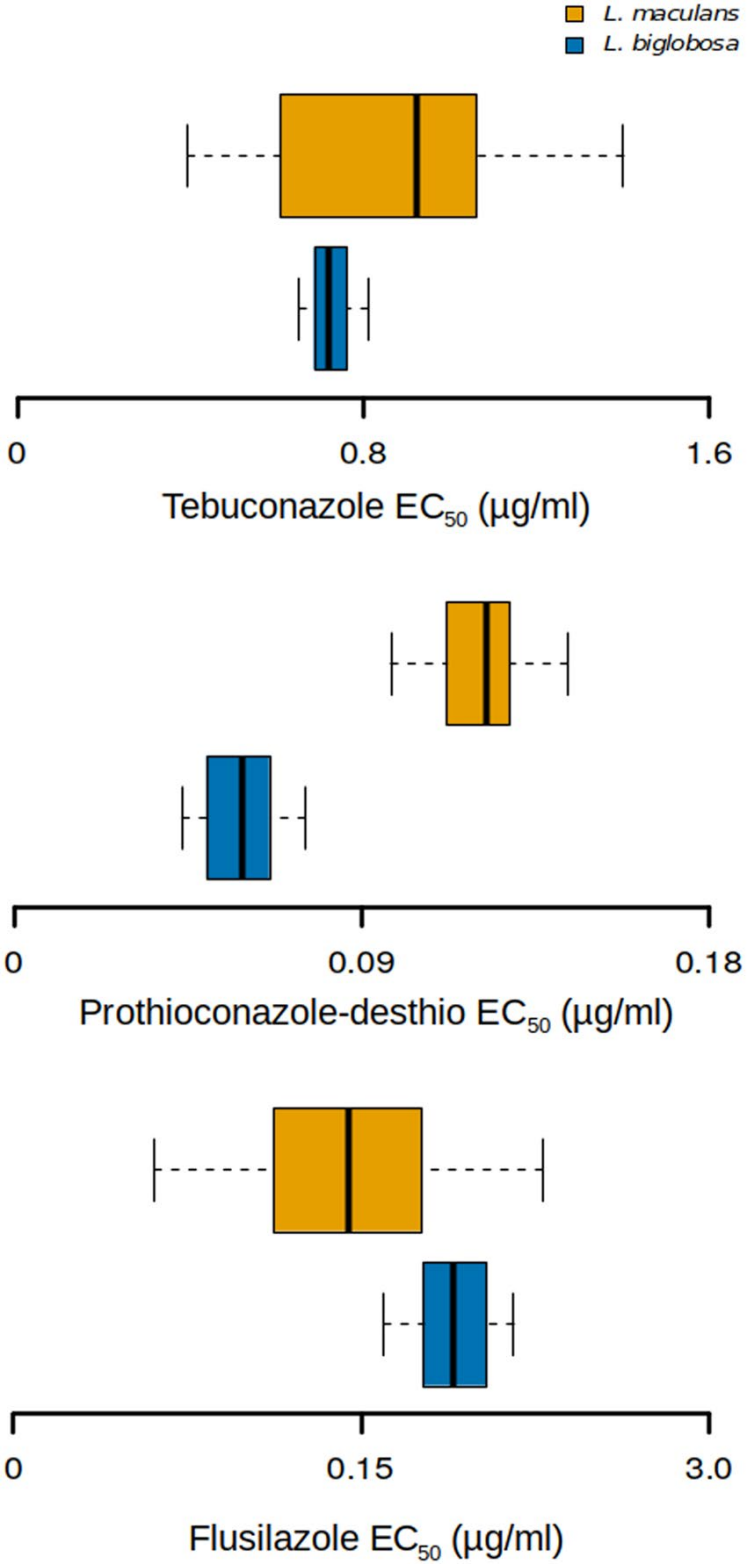
Box plot depicting sensitivities of *Leptosphaeria maculans* (yellow) isolates (n = 23) and *L. biglobosa* (blue) isolates (n = 21) to flusilazole (**a**), prothioconazole-desthio (**b**) and tebuconazole (**c**). Thick line denotes median value, box edges signify lower and upper quartiles and error bars represent minimum and maximum values.

Both species were very sensitive to prothioconazole-desthio, flusilazole and tebuconazole (Figs 5 and ST 1). Comparison between *L. maculans* (n = 23) and *L. biglobosa* (n = 21) isolates identified a significant difference in sensitivity to flusilazole (Fig. 5a) (W = 373, *P* < 0.01) and prothioconazole-desthio (Fig. 5b) (W = 39, *P* < 0.001). *L. maculans* isolates were 2.07 times less sensitive than *L. biglobosa* isolates to prothioconazole desthio, whereas *L. biglobosa* was 1.31 times less sensitive to flusilazole than *L. maculans*. There was no significant difference in sensitivity to tebuconazole (Fig. 5c).

### Prothioconazole sensitivity *in planta*

**Figure 6:**
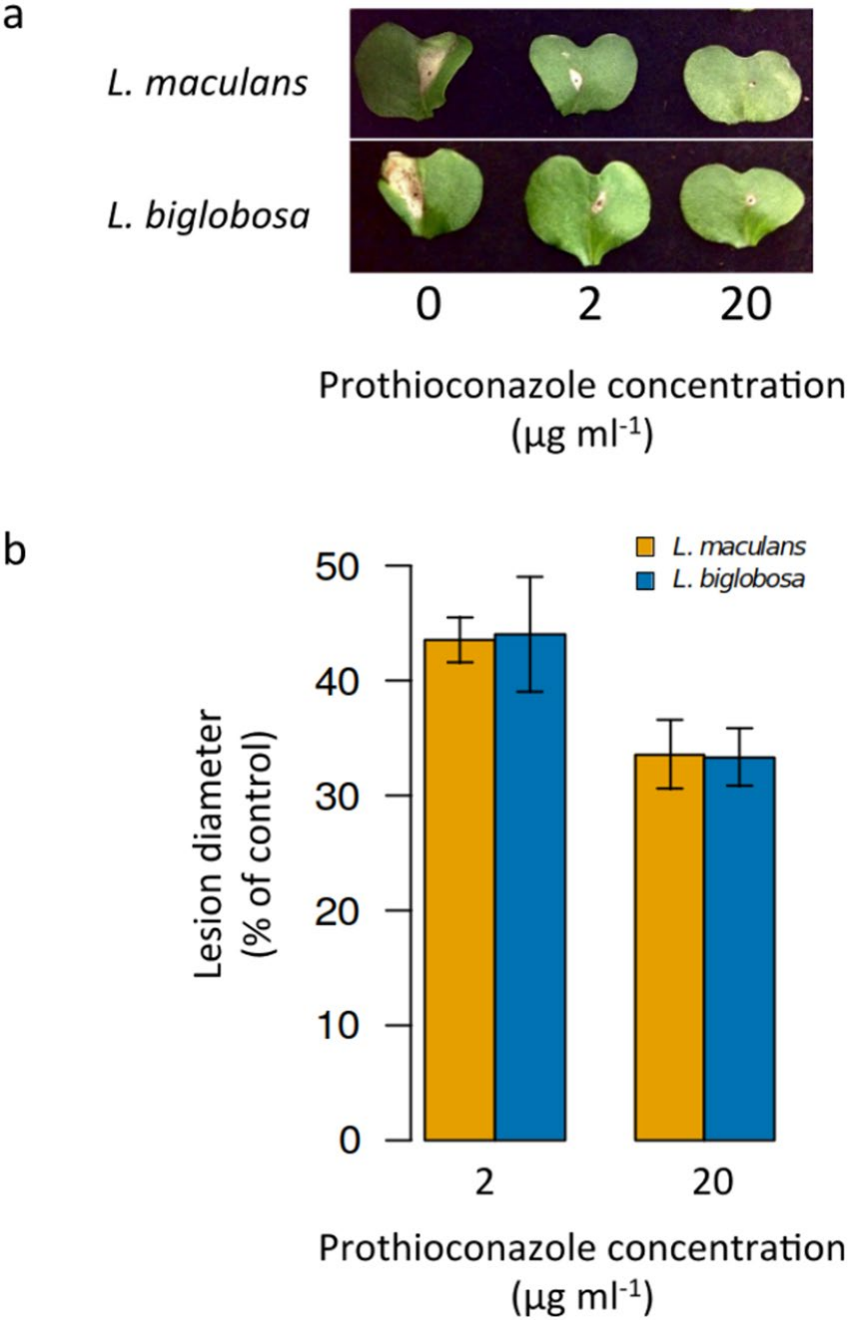
Efficacy of prothioconazole on the leaf lesions of *Leptosphaeria maculans* (yellow) and *L. biglobosa* (blue). Oilseed rape cotyledons (cv. Catana) were inoculated with either *L. maculans* or *L. biglobosa* conidial suspension (×10^6^). At 6 days post inoculation (DPI), cotyledons were sprayed with prothioconazole at 0 µg ml^−1^, 2 µg ml^−1^ or 20 µg ml^−1^ concentration (**a**). Lesion diameter (mm) was recorded and average lesion diameter calculated as percentage compared to control (**b**). Error bars represent standard errors of the mean.

At 14 days post inoculation (DPI), treatment with prothioconazole showed a significant effect on *L. maculans* or *L. biglobosa* lesion diameter (Kruskal-Wallis chi-squared = 124.4, 2 df, *P* < 0.05) (Fig. 6), with lesion diameter decreasing as concentration increased. A concentration of 20 µg ml^−1^ prothioconazole reduced both *L. maculans* and *L. biglobosa* lesion size by three-fold compared to the untreated control. Moreover, *L. biglobosa* and *L. maculans* lesions were not statistically different in their size on prothioconazole-treated leaves.

**Figure 7:**
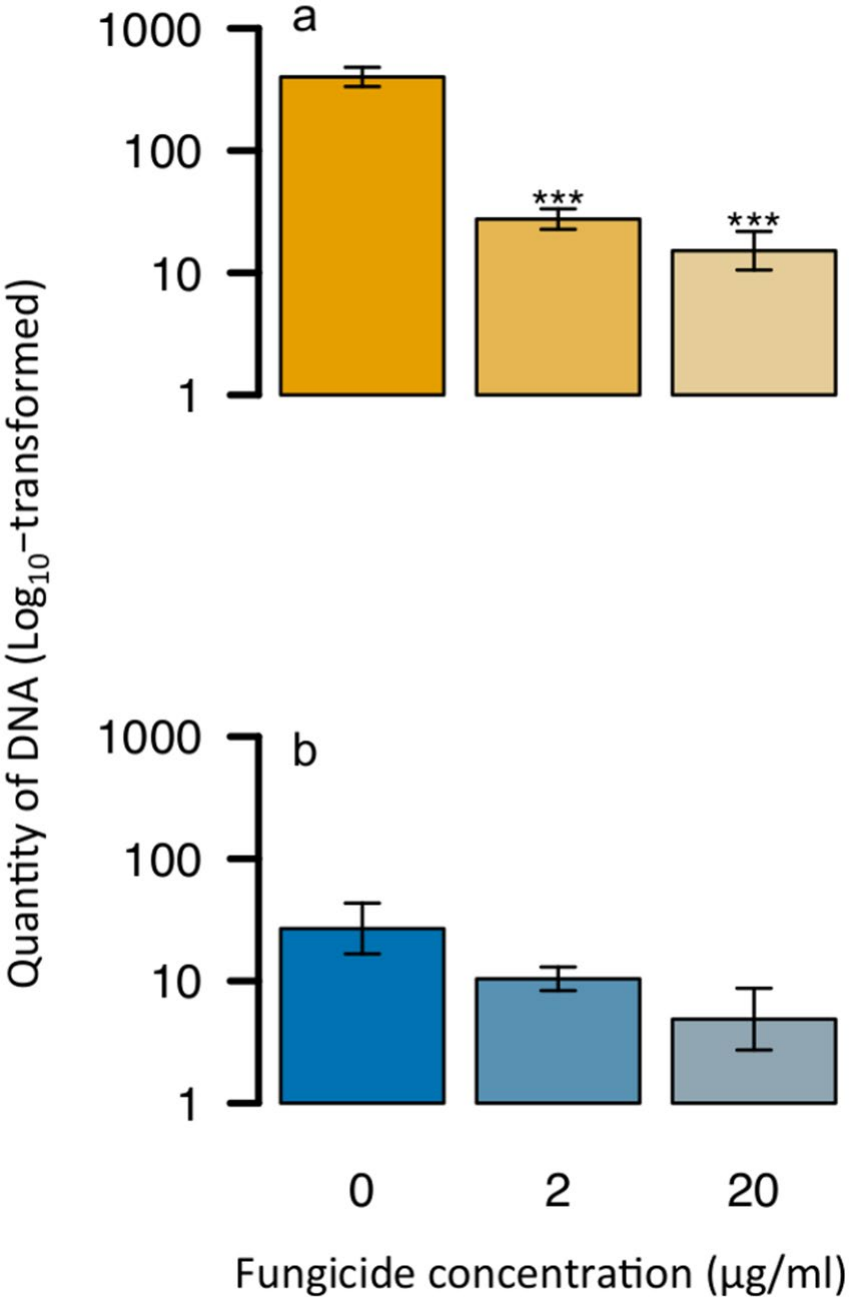
Quantification of DNA from prothioconazole-treated oilseed rape cotyledons inoculated with either *L. maculans* (**a**) or *L. biglobosa* (**b**). Oilseed rape cotyledons (cv. Catana) were inoculated with either *L. maculans* or *L. biglobosa* conidial suspension (×10^6^ conidia ml^−1^). At 6 days post inoculation (DPI), cotyledons were sprayed with prothioconazole at either 0 µg ml^−1^, 2 µg ml^−1^ or 20 µg ml^−1^ concentration. Cotyledons were sampled at 14 DPI and DNA was extracted (25 ng μl^−1^). Quantitative PCR was done on duplicate samples and run alongside a standard curve for quantification of DNA (pg ul^−1^). Data were log_10_-transformed. Asterisks denote significance when compared with the control (*P < 0.05, **P < 0.01 ***P < 0.001) and error bars represent standard deviation.

DNA quantity for each treatment was determined using qPCR to measure the effect prothioconazole treatment has on pathogen growth (Fig. 7) *in planta*. Both 2 and 20 µg ml^−1^ of prothioconazole significantly decreased the amount of *L. maculans* DNA in leaf tissue when compared to the untreated control (2 df, P < 0.05); however, there was no significant difference in DNA quantity between 2 and 20 µg ml^−1^. There was no significant difference in the amount of *L. biglobosa* DNA within leaf tissues treated across all three concentrations (0, 2, 20 µg ml^−1^).

## Discussion

Heterologous expression of lanosterol 14α-demethylase (CYP51B) from *Leptosphaeria maculans* (LmCYP51B) and *L. biglobosa* (LbCYP51B), together with subsequent fungicide sensitivity assays on the yeast transformants, show that LmCYP51B and LbCYP51B interact similarly with azole fungicides tebuconazole, flusilazole and prothioconazole-desthio. These observations support the homology modelling predictions that LmCYP51B and LbCYP51B share a high level of structural similarity, specifically at the azole-binding site.

Sequence analysis indicated that both LmCYP51B and LbCYP51B contained no previously identified CYP51B mutations that confer azole resistance or azole insensitivity in other plant and human pathogenic fungi^8,22–24^. Therefore, evolution of resistance to azole fungicides, which has been strongly linked to the gradual development of missense mutations in the CYP51B amino acid sequence^17,18,20,25,26^, has not yet occurred in either the *L. maculans* or the *L. biglobosa* populations sampled. Nonetheless, further analysis was warranted due to multiple amino acid substitutions between LmCYP51B and LbCYP51B, plus two extra residues in LbCYP51, that could potentially affect the tertiary structure of the proteins and thus the azole binding potential.

Changes to the structure of CYP51B have previously been proposed to alter the affinity of azoles, such as tebuconazole or triadimenol; this was determined through predictive homology modelling of wild type (MgCYP51B) and mutant CYP51B proteins found in insensitive *Mycosphaerella graminicola* populations^20^. More recently, analysis of a high resolution *S. cerevisiae* CYP51B structure (PDB:4WMZ), co-crystallised with medical azole fluconazole, identified two water molecules within the active site of the proteins that form a network of hydrogen bonds adhering the azole to the protein^27^. The presence of Y136F/H substitution (ScCYP51B) breaks one part of this water-mediated hydrogen bond network due to the loss of the hydroxyl group incurred by the tyrosine to phenylalanine or histidine substitution. This reduces the azole binding capacity of the proteins, confirmed through spectral characterisation of fluconazole binding with the wildtype ScCYP51B protein or Y140F/H mutants^28^.

Here the predicted structures of LmCYP51B and LbCYP51B have been compared through the structural alignment of homology models. Prothioconazole-desthio, the more active *in planta* metabolite of agricultural azole prothioconazole^29^, was superimposed using its confirmation in co-crystallised ScCYP51B structure PDB:5EAD. Analysis of the alignment (Fig. 3) noticeably illustrates that structural conservation is maintained throughout the globular pocket. Only in the transmembrane tail is there any noticeable distance between the aligned residues. Locality of residues: Y136, K147 and *G461/G462 (*LmCYP51B/LbCYP51B, respectively), which correspond to substitutions Y132F, K143R and G464S in resistant *Candida albicans* isolates (Y140F, K151R and G464S in ScCYP51B)^24^, are all conserved in *L. maculans* and *L. biglobosa*. In addition to this, Y122 (Y126 in ScCYP51B) and *S374/S375 (S383 in ScCYP51B), which form a water-mediated hydrogen bond network with another water molecule when fluconazole is bound, are also structurally conserved.

Consequentially, it can be postulated that the wild type LmCYP51B and LbCYP51B proteins would interact with azole fungicides similarly due to the likeness of key structural locations and the preservation of a water-mediated hydrogen bond network in the predicted homology models. This hypothesis was tested using heterologous yeast expression and subsequent fungicide sensitivity assays, to investigate the efficacy of various azole fungicides on LmCYP51B and LbCYP51B within an isogenic background; previous studies have used this method to investigate the effects of CYP51B mutations on fungicide efficacy, showing a causal link between key mutations and resistant phenotypes^16,17,26^. Both LmCYP51B and LbCYP51B yeast transformants responded similarly to azole treatment, suggesting that the affinity of various azole fungicides to both LmCYP51B and LbCYP51B is similar, with the only significant difference between the two transformants a 1.35-fold change in tebuconazole EC_50_. This represents a minor and possibly non-effective difference in the field, especially when compared to MgCYP51B harbouring mutations L50S and Y461H, which conferred a resistance factor (fold-change against wild-type) of 63.2 to tebuconazole and 192 to triadimenol, when expressed in yeast^26^.

Furthermore, this small difference in sensitivity of the heterologously-expressed proteins was not observed at the organism level, where both *L. maculans* and *L. biglobosa* isolates had a similar response to tebuconazole *in vitro*. Interestingly, the two species did differ in their sensitivity to flusilazole (2.07 fold change) and prothioconazole-desthio (1.31 fold change), both of which did not exhibit a differing effect on LmCYP51B and LbCYP51B when heterologously expressed in yeast. It is possible that these minor differences in sensitivity could be due to natural variation in the uptake, efflux or metabolism of fungicide compounds, however to determine this would require further investigation.

Ultimately, all *L. maculans* and *L. biglobosa* isolates sampled were sensitive to tebuconazole, flusilazole and prothioconazole-desthio. The distribution of EC_50_ values for each fungicide were small, suggesting that no isolate in our collection had developed increased resistance to fungicides. However, our data set provides only an example of intrinsic sensitivity of the two species from a mechanistic perspective and does not rule out sensitivity variation in the wider population. Nonetheless, on-going field studies conducted on a yearly basis in the UK by the Agricultural and Horticultural Board, have yet to identify a shift in sensitivity by either *Leptosphaeria* spp, with fungicides remaining effective for the control of phoma stem canker^30^.

These *in vitro* sensitivity data, together with CYP51B sequence analysis, are in line with previous studies that found no evidence of azole resistance among populations of either *L. maculans* or *L. biglobosa* ^11,12^. Some similarities in sensitivity are observed *in planta*, with prothioconazole treatment having comparable effects on *L. maculans* and *L. biglobosa* lesion size when colonising oilseed rape cotyledons. However, only *L. maculans* DNA quantity was significantly affected by 2–20 ng ml^−1^ prothioconazole, suggesting that *L. biglobosa* may have a minor difference in sensitivity to prothioconazole treatment *in plant*a or maybe less virulent on the tested cultivar since DNA quantity was much lower when compared to *L. maculans*. Further work using a wider range of *L. biglobosa* and *L. maculans* isolates is needed before conclusions on *in planta* sensitivity differences can be made. Overall, in light of just minor sensitivity differences both *in planta* and *in vitro*, changes in species frequency due to fungicide application, previously reported in other plant pathogens such as *Tapesia* spp.^13^, are unlikely to be the reason behind recent reports of increased *L. biglobosa* incidence^14^. Other factors, such as climatic pressures^31,32^, varietal specificity^33,34^ or even other anthropological influences such as pollution level^35^ could explain these frequency alterations and should be prioritised for further investigation.

Fungicides remain an essential component of integrated disease management and improved understanding of their efficacy on crop diseases is imperative. Knowledge that *L. maculans* and *L. biglobosa* have yet to evolve resistance to the azole fungicides tested here, reinforces confidence in the durability of these chemicals for the management of phoma stem canker and their suitability as mixing partners for higher risk fungicide groups. This knowledge, coupled with evidence suggesting that there is no great difference in azole sensitivity between the two species, suggests that currently neither pathogen will be actively selected through the prolonged usage of these fungicides. Further investigation into the potential future evolution of fungicide resistance of *L. maculans* and *L. biglobosa*, through the generation of laboratory mutants, will help towards enhancing the longevity of fungicides for future control of these pathogens. Furthermore, additional analysis into *L. maculans* and *L. biglobosa* frequency fluctuations, and the importance of *L. biglobosa* to UK oilseed rape production, is essential, but with a focus on environmental and varietal selection pressures.

## Methods

### Characterisation of *erg11* and CYP51B in *L. biglobosa*

*L. biglobosa* CYP51B (LbCYP51B) characterisation began with a BLASTp search of GenBank using the published *L. maculans* CYP51B (LmCYP51B) protein sequence (AAN28927.1). A Clustal Omega multiple sequence alignment was generated using six protein sequences with similar percentage identity: *Pyrenophora tritici-repentis* (XP_001939023.1), *Alternaria alternata* (OAG21175.1)*, Stagonosporopsis caricae* (AMM76225.1)*, Botrytis cinerea* (CCD54835.1)*, Sclerotinia sclerotiorum* (XP_001594997.1) and *Aspergillus nidulans* (XP_681552.1)^36,37^. Using the alignment, a 13 amino acid region (DVVYDCPNSKLME) conserved across five of the six aligned species was selected for a custom BLASTp search using Geneious R9.1.4 (Biomatters Limited, New Zealand), which was executed against a database of translated open reading frames generated from published *L. biglobosa* genome data provided by INRA-Bioger, France^38^. The corresponding nucleotide sequence was manually identified using an annotations text search; BLASTn search was performed to corroborate the putative LbCYP51 nucleotide sequence.

### LmCYP51B and LbCYP51B sequencing

Total DNA from 12 *L. maculans* isolates and eight *L. biglobosa* isolates (ST 1) was extracted using DNAMITE Plant DNA extraction kit (Microzone) according to manufacturer’s specifications. PCR amplification was completed using sequencing primers for both species (ST 2) and REDTaq DNA polymerase (Sigma). Amplification conditions for both species were 1 cycle at 95 °C for 2 min; 35 cycles at 95 °C for 40 sec, 53.5 °C for 30 sec and 72 °C for 1 min; followed by a final extension at 72 °C for 5 min. PCR products were purified with a QIAquick PCR Purification kit (Qiagen, USA) according to the manufacturer’s guidelines. For sequencing, samples were prepared to a final concentration of 20–30 ng µl^−1^ and sent to GATC Biotech for Sanger sequencing; sequence data were analysed using Geneious R9.1.4 and contigs assembled using Cap3^39^, with low quality ends trimmed manually. Constructed contigs were aligned with the published LmCYP51B gene sequence (AY142146) as the reference sequence. Sequences were manually trimmed to the start (ATG) and stop codon (TAG). The recognised location of a single intron in LmCYP51B was removed manually. The predicted location of a single intron in LbCYP51B, inferred from the alignment with *L. maculans* reference sequence, was also removed. Finally, nucleotide sequences were checked using ORF Finder (NCBI 2016) and translated into amino acid sequence using Geneious R9.14.

Phylogenetic analysis was conducted using Molecular Evolutionary Genetics Analysis (MEGA7) software^40^. Firstly, CYP51A/B/C orthologs were obtained using a BLASTn search of GenBank with the published LmCYP51B nucleotide coding sequence from *L. maculans* and the CYP51A/C nucleotide coding sequences from *Fusarium graminearum*. Sequences were translated and a Clustal Omega alignment performed on the translated CYP51B protein sequences from *L. maculans* CYP51B, *L. biglobosa* CYP51B, *P. tritici-repentis* CYP51B, *A. alternata* CYP51B*, Phaeosphaeria nodorum* CYP51B (XM_001938988.1)*, Stagonosporopsis cucurbitacearum* CYP51B (ANP43771.1), *B. cinerea* CYP51B, *S. sclerotiorum* CYP51B, *F. graminearum* CYP51B (FJ216402.1)*, A. fumigatus* CYP51B (XM_744041.1), *A. flavus* CYP51B (KOC13803.1)*, A. niger* CYP51B (GAQ46773.1), *F. graminearum* CYP51A (JN41662.1), *A. fumigatus* CYP51A (JX283445.1), *F. graminearum* CYP51C (AGG55728.1) and *Saccharomyces cerevisiae* CYP51 (NP_011817.1), with *Homo sapiens* CYP51 (NP_000777.1) as an outgroup. In order to identify potential CYP51 paralogs in both *Leptopshaeria* spp., a tBLASTn search was performed on the two *Leptopshaeria* spp. genomes using *F. graminearum* CYP51A/C protein sequences and the *S. cerevisae* CYP51 sequence. The closest match that was not CYP51B was included in the alignment and subsequent phylogeny. A codon alignment was used for model selection through maximum-likelihood (BIC) and then phylogenetic relationships were inferred by maximum-likelihood using the general time reversible nucleotide substitution model^41^ with 1000 bootstrap replications.

### Comparative homology modelling

Predictive homology protein modelling was processed in Chimera^42^ using Modeller v9.14^43^. Firstly, to identify similar CYP51B sequences that have been structurally solved, a BLASTp search of the protein data bank (PDB) database was done individually on LmCYP51B and LbCYP51B amino acid sequences.

A Clustal Omega multiple sequence alignment was performed on the three most homologous CYP51B protein sequences, together with either LmCYP51B or LbCYP51B. For both *Leptosphaeria* spp., the closest solved CYP51B structures were from *A. fumigatus, S. cerevisiae* and *H. sapiens* (SF 1a-b). In Chimeria, bound ligands within these structures were removed and the heme cofactor, which is an important feature of CYP51B, was manually removed from the *S. cerevisiae* and *H. sapiens* structures but conserved from *A. fumigatus*, as this was the protein with the highest sequence identity.

The Advanced options in Modeller v9.14 were used to build the model with hydrogens and the number of output models was set to five. Selection of the most reliable model was done using ModFOLD4^44^. Prothioconazole-desthio, in the PDB: 5EAD binding confirmation was superimposed into the predicted structures using Matchmaker in Chimera with parameters set to default. Hydrogen bonds were predicted using H-bond finder in Chimera with parameters set to default. A structural alignment (TM-align) resulting from a residue-to-residue comparison was generated with a 0 to 1 scoring system where 1 indicates a perfect match between the two structures^45^.

### Heterologous expression of LmCYP51 and LbCYP5 in *Saccharomyces cerevisiae*

Total RNA was extracted from one *L. maculans* (Hrox 12-2-1) and one *L. biglobosa* (F_2_ dm 11-5) isolate using the RNeasy Plant Mini Kit (Qiagen). Mycelium of each species was grown in Sabouraud dextrose broth for 10 days, harvested through centrifugation, flash-frozen in liquid nitrogen, freeze-dried for 24 h and then ground into a fine powder. The remaining RNA extraction procedure was done according to manufacturer’s instructions. Complementary DNA (cDNA) was synthesised from 1 µg of total RNA using the SuperScript® IV First-Strand Synthesis System (SSIV) (Invitrogen) according to manufacturer’s guidelines. The complete LmCYP51B and LbCYP51B coding sequences were amplified using primer pairs LmCYP51res (forward/reverse) and LbCYP51res (forward/reverse) (ST 2). Restriction primers introduced a *HindIII* restriction site to the 5″ end and a *NotI* restriction site to the 3″ of the amplified CYP51B sequences.

A touchdown-PCR^46^ was completed using EasyA High Fidelity PCR Cloning Enzyme (Agilent, USA). Amplification conditions for LmCYP51 were 1 cycle at 94 °C for 2 min; 7 cycles at 94 °C for 30 sec, 59 to 53 °C each for 1 min (1 °C decrease per cycle) and 72 °C for 2 min; 28 cycles at 94 °C for 30 sec, 52 °C for 1 min and 72 °C for 1 min; followed by a final extension at 72 °C for 7 min. Amplification conditions for LbCYP51 were 1 cycle at 94 °C for 2 min; 7 cycles at 94 °C for 30 sec, 58 to 52 °C each for 1 min (1 °C decrease per cycle) and 72 °C for 2 min; 28 cycles at 94 °C for 30 sec, 56 °C for 1 min and 72 °C for 1 min; followed by a final extension at 72 °C for 7 min. PCR products were purified, cloned into the pGEM-T easy vector (Promega, USA) and transformed into high efficiency JM109 competent cells (Promega) using ampicillin selection and blue-white screening, all according to the manufacturer’s specification. Colony PCR used primers LmCYP51res (forward/reverse) and LbCYP51res (forward/reverse) to determine successful ligation of LmCYP51B or LbCYP51B. PCR was carried out using REDTaq DNA polymerase (Sigma) with a 52 °C or 51 °C annealing temperature, respectively, and 40 seconds extension time. Plasmids were extracted using the QIAprep Spin Miniprep Kit (Qiagen).

Restriction digestions of pGEM-T-LmCYP51 and pGEM-T-LbCYP51 constructs were prepared using *HindIII* and *NotI* high fidelity enzymes (New England Biolabs) according to manufacturer’s double-digestion specification. The yeast vector, pYES2/CT (Thermo Fisher, USA) was digested under the same conditions. Digested pYES2/CT, LmCYP51 and LbCYP51 were purified by gel electrophoresis (0.7% agarose) using QIAquick gel extraction kit according to manufacturer’s specification. Purified samples were then ligated and transformed into high efficiency JM109 competent *E. coli* cells. Colony PCR was used to check which *E. coli* colonies had the correct insertion. Positive transformants underwent plasmid extraction and the purified pDNA was used for transformation of *S. cerevisiae* strain YUG37: *erg11* (MATa ura3-52 trp1-63 LEU2::tTA tetO- CYC1::erg11)^47^ using the S.c EasyComp transformation kit (Invitrogen, USA). YUG37:LmCYP51B and YUG37:LbCYP51B transformants were plated out onto synthetic dropout minimal medium (SD) containing 4 g l^−1^ yeast nitrogen base without amino acids (Sigma), 3.92 g l^−1^ dropout medium supplement without uracil (Sigma), 2% galactose (GAL) and 2% raffinose (RAF).

YUG37:LmCYP51B, YUG37:LbCYP51B and YUG37:pYES2/CT (negative control) transformants were grown in 10 ml liquid SD medium for 24 h at 30 °C with shaking (250 RPM). Cell suspensions were diluted to a concentration of 10^6^ cells ml^−1^ and inoculated onto SD GAL + RAF agar plates with or without 3 µg ml^−1^ doxycycline (5 µl of five 5-fold dilutions). Plates were incubated at 30 °C and photographed after 5 days.

### Fungicide sensitivity of yeast transfomants

Fungicide sensitivity of YUG37:LmCYP51B and YUG37:LbCYP51B transformants was tested using flusilazole, tebuconazole and prothioconazole-desthio fungicides (2 independent transformants per protein). A single transformant colony was grown in 10 ml liquid SD GAL + RAF for 24 h at 30 °C with shaking. The resultant cell suspensions were diluted to a concentration of 10^6^ cells ml^−1^ and 100 µl aliquots were added to a flat-bottomed 96-well microtitre plate. The microtitre plate was subsequently populated with 100 µl of SD GAL + RAF media with doxycycline (6 µg ml^−1^), which had been amended with decreasing concentrations of fungicide (5, 1.7, 0.56, 0.19, 0.062, 0.021, 0.0069, 0.0023, 0.00076, 0.00025, 8.47 × 10^−5^, 2.82 × 10^−5^, 0 µg ml^−1^). Plates were incubated at 30 °C for 6 days. Fungal growth was measured by absorbance using a FLUOstar OPTIMA microplate reader (BMG Labtech, Offenburg, Germany) set to 630 nm in endpoint mode. Fungicide sensitivities for each isolate were calculated as 50% effective concentrations (EC_50_) using a dose-response relationship curve generated by the FLUOstar OPTIMA microplate software. Distribution of data was determined using a Shapiro-Wilk normality test in R^48^. Significant differences in the distribution of EC_50_ values between species were determined using a One-Way ANOVA in R.

### Fungicide sensitivity *in vitro*

Sensitivity assays, modified from those of Pijls *et al*.^49^, consisted of 2x sabouraud dextrose media amended with decreasing concentrations of technical grade flusilazole, tebuconazole (20, 10, 5, 2.5, 1.25, 0.63, 0.31, 0.16, 0.078, 0.039, 0.020, 0 µg ml^−1^ final concentration) or prothioconazole-desthio (20, 6.67, 2.22, 0.74, 0.25, 0.082, 0.027, 0.009, 0.003, 0.001, 0.0003, 0 µg ml^−1^ final concentration). Aliquots (100 µl) of the fungicide-amended media were added to the wells of flat-bottomed 96-well microtitre plates (NUNC™, Thermo Fisher, USA). Aliqouts (100 µl) of individual conidial suspensions (10^6^ conidia ml^−1^) of 23 *L. maculans* isolates and 22 *L. biglobosa* isolates (isolated between 2014 and 2015) were added to each well of a single row (in duplicate). Plates were incubated at 20 °C for 4 days and fungal growth measured by absorbance using a FLUOstar OPTIMA microplate reader, as previously described. Distribution of data was determined using a Shapiro-Wilk normality test and significant differences in the distribution of EC_50_ values between species were determined using a Mann-Whitney *U*-test in R.

### Prothioconazole sensitivity *in planta*

Seeds of *B. napus*, cv. Catana (Dekalb, Monsanto, UK), were pre-germinated in a shallow rectangular germination tray (50 × 30 × 8 cm) containing a mixture of 50% enriched general-purpose compost (Miracle-Gro, UK) and 50% John Innes No 3 compost (JA Bower, UK). After 10 days growth, plants were arranged in an alternate block design (two replicate blocks each containing six treatments with eight plants per treatment) located in a plant growth chamber (Conviron Europe, UK). The chamber was set to a 12-hour light/12-hour dark cycle with a light intensity of 210 μEm^2^ s^−1^. Under regular growing conditions, relative humidity in the chamber was set at 70% and temperature was set at 20 °C when light and 18 °C when dark.

After 12 days growth, the cotyledons were inoculated with the conidial suspension of one *L. maculans* (H_ROX_ 12-2-1) or one *L. biglobosa* (F_2_ Exc dm 11-5) isolate by pin-hole wounding^50^. Conidial suspension (10 µl), at a concentration of 10^6^ conidia ml^−1^, was applied directly to each wound site. After inoculation, the plants were left for 24 hours in darkness and 100% humidity, before re-establishing normal growing conditions. At 6 days post inoculation (DPI), commercial grade prothioconazole was applied at a concentration of 2 µg ml^−1^ or 20 µg ml^−1^ using a water atomiser. Untreated control (0 µg ml^−1^ prothioconazole) cotyledons were sprayed with water.

Disease assessments were completed by measuring lesion diameter (mm) at 14 DPI. The cotyledons were subsequently removed, instantly frozen in liquid nitrogen and stored at −80 °C for qPCR analysis. Frozen leaves were ground into a fine powder and DNA was extracted and diluted to a concentration of 25 ng μl^−1^ (starting concentration). Amplification of *L. maculans* or *L. biglobosa* DNA was done using LmacF/LmacR or LbigF/LmacR primers, respectively. The PCR reactions used Brilliant III Ultra-fast SYBR Green qPCR master mix with low Rox at a final volume of 20 μl and a primer concentration of 0.3 μM. PCR parameters were as follows: initial denaturation at 95 °C for 2 min, followed by 40 cycles of 95 °C for 15 sec, 60 °C (*L. maculans*) or 55 °C (*L. biglobosa*) for 30 sec and 72 °C for 36 sec.

Distribution of data was determined using a Shapiro-Wilk normality test in R. Significant differences in lesion sizes between species and between fungicide concentrations were determined independently using a Kruskal-Wallis rank sum test in R. Significant differences in DNA quantity between species were determined using One-Way ANOVA and *post hoc* Tukey honest significant test in R.

## Electronic supplementary material

Supplementary figures and tables
