# Supplementary material for: Azole sensitivity in Leptosphaeria pathogens of oilseed rape: the role of lanosterol 14α-demethylase
Source: Sci Rep. 2017 Nov 20;7:15849. doi: 10.1038/s41598-017-15545-9 (PMC5696480; doi:10.1038/s41598-017-15545-9)
Supplement: Supplementary file 1 — Supplementary figures and tables [file 41598_2017_15545_MOESM1_ESM.pdf]

Supplementary figures and tables

Azole sensitivity in Leptosphaeria pathogens of oilseed rape: the role of lanosterol 14α-demethylase.

Thomas R Sewell,
Department of Infectious Disease Epidemiology, Imperial College London, UK

Nichola J Hawkins,
Biointeractions and Crop Protection, Rothamsted Research, Harpenden, Hertfordshire, UK

Henrik U Stotz
Crop Protection Group, Centre for Agriculture, Food and Environmental Management, University of Hertfordshire, Hatfield, UK

YongJu Huang
Crop Protection Group, Centre for Agriculture, Food and Environmental Management, University of Hertfordshire, Hatfield, UK

Steven L Kelly
Centre for Cytochrome P450 Biodiversity, Institute of Life Science, School of Medicine, Swansea University, Swansea, UK

Diane E Kelly
Centre for Cytochrome P450 Biodiversity, Institute of Life Science, School of Medicine, Swansea University, Swansea, UK

Bart Fraaije
Biointeractions and Crop Protection, Rothamsted Research, Harpenden, Hertfordshire, UK

Bruce DL Fitt
Crop Protection Group, Centre for Agriculture, Food and Environmental Management, University of Hertfordshire, Hatfield, UK

ST 1: Table of isolates

Isolate	Species	Location	Year	Host crop	Cultivar	Isolation method	Flusilazole EC50 (µg/ml)	Tebuconazole (EC50) (µg/ml)	Prothioconazole-desthio (EC50) (µg/ml)	CYP51B sequenced
ME24	*L. maculans*	Yorkshire	2002	*Brassica napus*	Apex	Single pycnidium	0.18	0.92	0.12	
TS13-10	*L. maculans*	Cambridge, UK	2013	*Brassica napus*	Catana	Single pycnidium	0.08	0.60	0.12	x
TS13-11	*L. maculans*	Cambridge, UK	2013	*Brassica napus*	Catana	Single pycnidium	0.23	1.40	0.11	
TS13-12	*L. maculans*	Cambridge, UK	2013	*Brassica napus*	Catana	Single pycnidium	0.12	1.11	0.05	x
TS13-13	*L. maculans*	Cambridge, UK	2013	*Brassica napus*	Catana	Single pycnidium	0.16	1.08	0.13	x
TS13-19	*L. maculans*	Cambridge, UK	2013	*Brassica napus*	Catana	Single pycnidium	0.13	0.61	0.14	
TS13-2	*L. maculans*	Cambridge, UK	2013	*Brassica napus*	Catana	Single pycnidium	0.20	0.97	0.12	x
TS13-3	*L. maculans*	Cambridge, UK	2013	*Brassica napus*	Catana	Single pycnidium	0.37	1.89	0.11	
TS13-4	*L. maculans*	Cambridge, UK	2013	*Brassica napus*	Catana	Single pycnidium	0.14	0.85	0.13	x
TS13-5	*L. maculans*	Cambridge, UK	2013	*Brassica napus*	Catana	Single pycnidium	0.13	0.81	N/A	
TS13-6	*L. maculans*	Cambridge, UK	2013	*Brassica napus*	Catana	Single pycnidium	0.35	1.79	0.10	
TS13-7	*L. maculans*	Cambridge, UK	2013	*Brassica napus*	Catana	Single pycnidium	0.18	0.98	N/A	x
TS13-8	*L. maculans*	Cambridge, UK	2013	*Brassica napus*	Catana	Single pycnidium	0.08	0.48	N/A	
TS13-9	*L. maculans*	Cambridge, UK	2013	*Brassica napus*	Catana	Single pycnidium	N/A	N/A	0.11	
TS14-1	*L. maculans*	Cambridge, UK	2014	*Brassica napus*	Catana	Single pycnidium	0.10	0.59	0.12	x
TS14-12	*L. maculans*	Cambridge, UK	2014	*Brassica napus*	Catana	Single pycnidium	0.12	0.72	0.14	
TS14-13	*L. maculans*	Cambridge, UK	2014	*Brassica napus*	Catana	Single pycnidium	0.17	1.12	0.12	x
TS14-14	*L. maculans*	Cambridge, UK	2014	*Brassica napus*	Catana	Single pycnidium	0.12	0.62	0.12	
TS14-2	*L. maculans*	Cambridge, UK	2014	*Brassica napus*	Catana	Single pycnidium	0.10	0.61	0.13	x
TS14-21	*L. maculans*	Cambridge, UK	2014	*Brassica napus*	Catana	Single pycnidium	0.17	1.00	0.13	
TS14-4	*L. maculans*	Cambridge, UK	2014	*Brassica napus*	Catana	Single pycnidium	0.16	1.04	0.12	
TS14-9	*L. maculans*	Cambridge, UK	2014	*Brassica napus*	Catana	Single pycnidium	0.17	0.96	0.10	x
TS13-18	*L. maculans*	Cambridge, UK	2013	*Brassica napus*	Catana	Single pycnidium	0.11	0.45	0.10	x
H Rox 12-2-1	*L. maculans*	Cowlinge, UK	2012	*Brassica napus*	Roxet	Single pycnidium	0.06	0.39	0.13	x
B2003.2.8	*L. biglobosa*	Orston, UK	2003	*Brassica napus*	Recital	Single pycnidium	0.19	0.92	0.06	
A Exc 12-10	*L. biglobosa*	Banbury, UK	2012	*Brassica napus*	Excel	Single pycnidium	0.19	0.65	0.05	
D Rox 12-10	*L. biglobosa*	Banbury, UK	2012	*Brassica napus*	Roxet	Single pycnidium	0.20	0.70	0.04	x
E Exc 12-9-21	*L. biglobosa*	Bainton, UK	2013	*Brassica napus*	Excel	Single pycnidium	0.26	0.98	0.07	
F2 Exc 12-2-3	*L. biglobosa*	Stockbridge, UK	2013	*Brassica napus*	Excel	Single pycnidium	0.20	0.79	0.07	x
F2 Exc 12-3-1	*L. biglobosa*	Stockbridge, UK	2013	*Brassica napus*	Excel	Single pycnidium	0.19	0.72	0.05	
F2 Exc 12-6-1	*L. biglobosa*	Stockbridge, UK	2013	*Brassica napus*	Excel	Single pycnidium	0.17	0.70	0.08	x
F2 Exc dm 11-5	*L. biglobosa*	Stockbridge, UK	2012	*Brassica napus*	Excel	Single pycnidium	0.16	0.92	0.07	x
C Rox 12-8-1	*L. biglobosa*	Morley, UK	2013	*Brassica napus*	Roxet	Single pycnidium	0.18	0.69	0.05	
H 12-6	*L. biglobosa*	Cowlinge, UK	2013	*Brassica napus*	Drakkar	Single pycnidium	0.18	0.69	0.04	
H Dr 12-12	*L. biglobosa*	Cowlinge, UK	2013	*Brassica napus*	Drakkar	Single pycnidium	0.21	0.81	0.06	
H Dr 12-2	*L. biglobosa*	Cowlinge, UK	2013	*Brassica napus*	Drakkar	Single pycnidium	0.18	0.67	0.06	x
H Exc 12-12-1	*L. biglobosa*	Cowlinge, UK	2013	*Brassica napus*	Excel	Single pycnidium	0.21	0.75	0.06	x
H Exc 12-12-31	*L. biglobosa*	Cowlinge, UK	2013	*Brassica napus*	Excel	Single pycnidium	0.22	0.72	0.06	
H Exc 12-2-2	*L. biglobosa*	Cowlinge, UK	2013	*Brassica napus*	Excel	Single pycnidium	0.18	0.72	0.06	x
K Rox 12-2-1 bc	*L. biglobosa*	Harpenden, UK	2012	*Brassica napus*	Roxet	Single pycnidium	0.17	0.68	0.06	
K 12-33	*L. biglobosa*	Harpenden, UK	2013	*Brassica napus*	Drakkar	Single pycnidium	0.17	0.66	0.05	
K Exc 12-10-21	*L. biglobosa*	Harpenden, UK	2012	*Brassica napus*	Excel	Single pycnidium	0.19	0.69	0.05	x
K Exc 12-11	*L. biglobosa*	Harpenden, UK	2012	*Brassica napus*	Excel	Single pycnidium	0.21	0.76	0.05	
K Rox 12-6-31	*L. biglobosa*	Harpenden, UK	2013	*Brassica napus*	Roxet	Single pycnidium	0.19	0.76	0.07	
Lb68	*L. biglobosa*	Welford, UK	2002	*Brassica napus*	N/A	Single pycnidium	0.08	0.36	0.18	

ST 2: Table of primers

Primer name	Sequence (5" - 3")	Experiment
LmacF	CTTGCCCACCAATTGGATCCCCTA	*Leptosphaeria* spp. identification
LmacR	GCAAAATGTGCTGCGCTCCAGG	
L.bigF	ATCAGGGGATTGGTGTCAGCAGTTGA	
L.bigR	GCAAAATGTGCTGCGCTCCAGG	
Lmac1_F	ACGTTGGTTTGATGGTCCGA	*Leptosphaeria* spp. sequencing
Lmac1_R	CCTCTTGTGTGAGGCCGAAT	
Lmac2_F	CCACTTACCACGCCCGTATT	
Lmac2_R	AGATCATGTCGTGCGAGTCC	
Lmac_3_F	CCGCTCCTTCGACTCCAAAT	
Lmac_3_R	CACAAATCCAAGCACGAGCC	
Lbig1_F	GTCGTCAGTTAGTGGTGCGA	
Lbig1_R	TTGTTGCCCGTCGTATCCAA	
L.big_2_F	TCCTCAAGCGCCACAAATCT	
L.big_2_R	GGGGTTGACGAAGTGGGAAT	
L.big_3_F	CTCCTGCTGAAGAAGCCGAA	
L.big_3_R	GAGGAGGATCCAGGCGATTG	
L.big_4_F	CATGATCGCCCTCCTCATGG	
L.big_4_R	AAATAA ATTGACGCTCTACTCCACC	
LmCYP51res_F	GCAAAGCTTATGGCTGTTCTTGCTACCGT	*Leptosphaeria* spp. restriction cloning (*HindIII* & *NotI*)
LmCYP51res_R	TTAGCGGCCGCCTACTCGACCTTCTCCCTCC	
LbCYP51res_F	GCAAAGCTTATGGGTGTTCTTGCTACCATTG	
LbCYP51res_R	TTAGCGGCCGCCTACTCCACCTTTTCTCTGCGC	
M13F	TGTAAAACGACGGCCAGT	pGEM-T-easy sequencing primers
M13R	CAGGAAACAGCTATGACC	

SF 1. Protein sequence alignment (Clustal Omega) of the three most homologous CYP51B protein sequences that have been structurally solved, together with that of LmCYP51B (a) or LbCYP51B (b). A BLASTp search of the protein data bank database was done individually on LmCYP51B and LbCYP51B protein sequences. PDB code and percentage similarity (%) are given next to the species name.

SF 2. Wild type LmCYP51B and LbCYP51B pair-wise amino acid sequence alignment (Clustal Omega). CYP51B sequence was determined in 12 *L. maculans* isolates and eight *L. biglobosa* isolates. There were no alterations within species and 26 alterations between species (highlighted in red). Secondary structure annotation was determined using Chimera.

SF 3. Two-dimensional protein-ligand interaction schematic (LigPlot$^+$) demonstrating LmCYP51 (a) and LbCYP51 (b) in association with fungicide prothioconazole-desthio (5L9). Prothioconazole-desthio, CYP51B side chain (Tyr136) and heme cofactor are shown in ball-and-stick representation. Ligand-metal bonds coloured in purple, hydrogen bonds are shown as green dotted lines, while the red-spoked arcs represent protein residues involved in hydrophobic interactions with the ligand. Water molecules (HOH) are displayed in light blue.

a

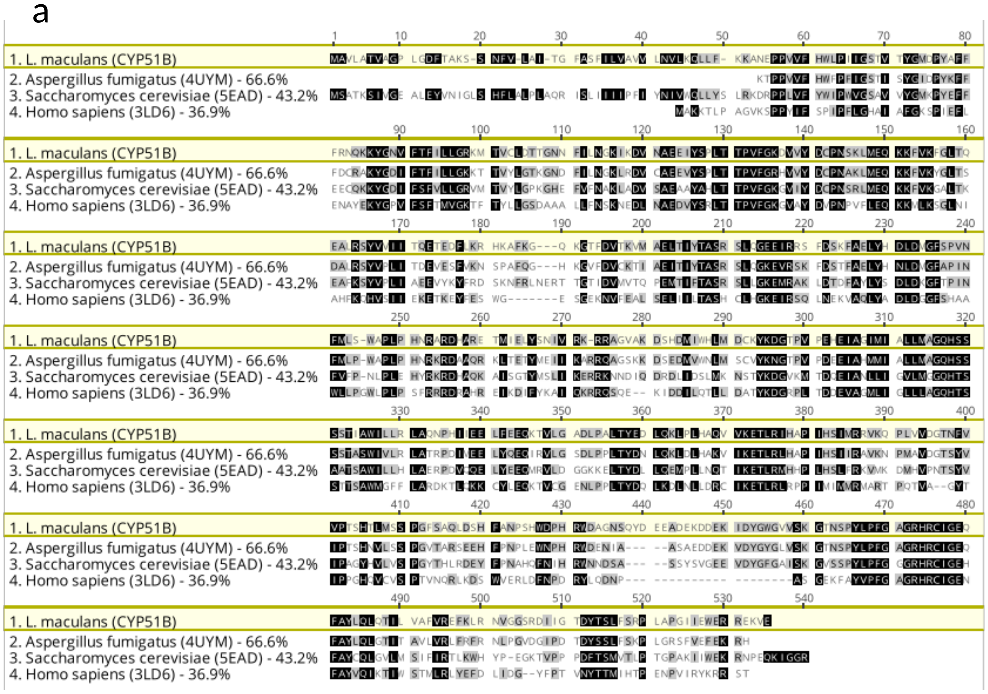
1. L. maculans (CYP51B)
2. Aspergillus fumigatus (4UYM) - 66.6%
3. Saccharomyces cerevisiae (5EAD) - 43.2%
4. Homo sapiens (3LD6) - 36.9%

b

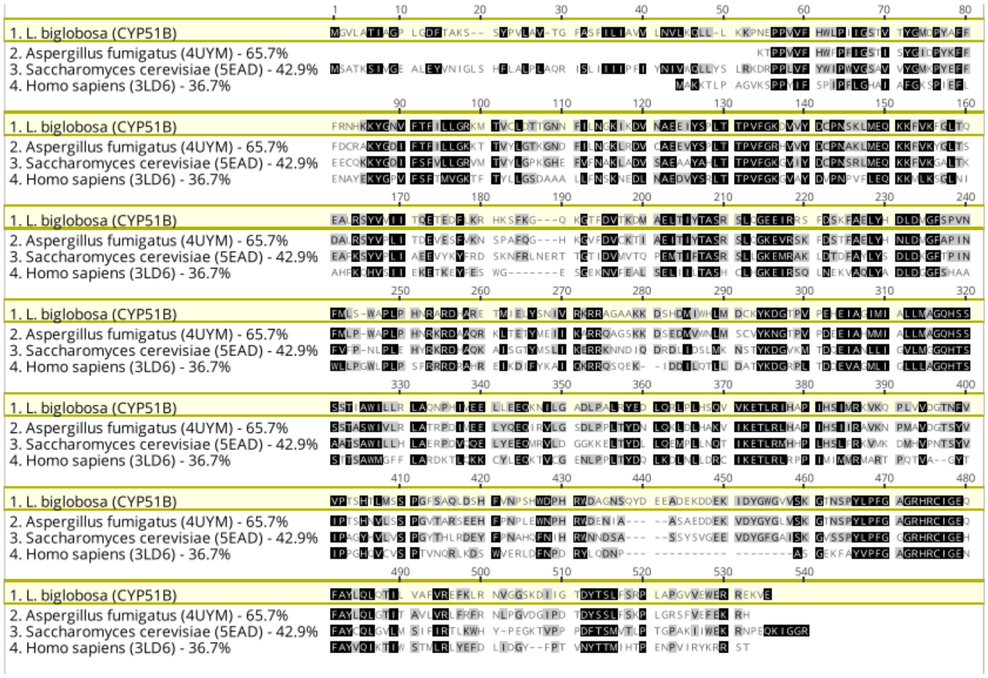
1. L. biglobosa (CYP51B)
2. Aspergillus fumigatus (4UYM) - 65.7%
3. Saccharomyces cerevisiae (5EAD) - 42.9%
4. Homo sapiens (3LD6) - 36.7%

SF 1

```
                 Lm-αA  Lm-αB
LmCYP51B    1  MAVLATVAGPLGDFTAKSSNFVLAITGFASFILVAVVLNVLKQLLFKKANEPPVVFHWLP   60
               M VLAT+AGPLGDFTAKSS  VLA+TGFASFIL+AVVLNVLKQLL KK NEPPVVFHWLP
LbCYP51B    1  MGVLATIAGPLGDFTAKSSYPVLAVTGFASFILIAVVLNVLKQLLLKKPNEPPVVFHWLP   60
                 Lm-αA  Lm-αB

                 Lm-αC  Lm-αD  Lm-β-1-1  Lm-β-1-2  Lm-αE
LmCYP51B   61  IIGSTVTYGMDPYAFFFRNQKKYGNVFTFILLGRKMTVCLDTTGNNFILNGKIKDVNAEE  120
               IIGSTVTYGMDPYAFFFRN KKYGNVFTFILLGRKMTVCLDTTGNNFILNGKIKDVNAEE
LbCYP51B   61  IIGSTVTYGMDPYAFFFRNHKKYGNVFTFILLGRKMTVCLDTTGNNFILNGKIKDVNAEE  120
                 Lm-αC  Lm-αD  Lm-β-1-1  Lm-β-1-2  Lm-αE

                 Lm-αF  Lm-αG  Lm-αH  Lm-αI
LmCYP51B  121  IYSPLTTPVFGKDVVYDCPNSKLMEQKKFVKFGLTQEALRSYVVIITQETEDFLKRHKAF  180
               IYSPLTTPVFGKDVVYDCPNSKLMEQKKFVKFGLTQEALRSYVVIITQETEDFLKRHK+F
LbCYP51B  121  IYSPLTTPVFGKDVVYDCPNSKLMEQKKFVKFGLTQEALRSYVVIITQETEDFLKRHKSF  180
                 Lm-αF  Lm-αG  Lm-αH  Lm-αI

                 Lm-β-2-1  Lm-αJ  Lm-αK  Lm-αL
LmCYP51B  181  KGQKGTFDVTKVMAELTIYTASRSLQGEEIRRSFDSKFAELYHDLDMGFSPVNFMLSWAP  240
               KGQKGTFDVTK MAELTIYTASRSLQGEEIRRSFDSKFAELYHDLDMGFSPVNFMLSWAP
LbCYP51B  181  KGQKGTFDVTKDMAELTIYTASRSLQGEEIRRSFDSKFAELYHDLDMGFSPVNFMLSWAP  240
                 Lm-β-2-1  Lm-αJ  Lm-αK  Lm-αL  Lm-αM

                 Lm-αM  Lm-αN
LmCYP51B  241  LPHNRARDHARETMIELYSNIVRKRRAGVAK-DSHDMIWHLMDCKYKDGTPVPEHEIAGI  299
               LPHNRARDHARETMIELYSNIVRKRRAG AK DSHDMIWHLMDCKYKDGTPVPEHEIAGI
LbCYP51B  241  LPHNRARDHARETMIELYSNIVRKRRAGAAKKDSHDMIWHLMDCKYKDGTPVPEHEIAGI  300
                 Lm-αN  Lm-αO

                 Lm-αO  Lm-αP  Lm-αQ
LmCYP51B  300  MIALLMAGQHSSSSTIAWILLRLAQNPHIIEELFEEQKTVLGADLPALTYEDLQKLPLHA  359
               MIALLMAGQHSSSSTIAWILLRLAQNPHI+EEL EEQK +LGADLPAL YEDLQ+LPLH+
LbCYP51B  301  MIALLMAGQHSSSSTIAWILLRLAQNPHIVEELLEEQKNILGADLPALRYEDLQRLPLHS  360
                 Lm-αP  Lm-αQ  Lm-αR

                 Lm-αR  Lm-β-3-1  Lm-β-1-2  Lm-αS
LmCYP51B  360  QVVKETLRIHAPIHSIMRRVKQPLVVDGTNFVVPTSHTLMSSPGFSAQLDSHFANPSHWD  419
               QVVKETLRIHAPIHSIMR+VKQPLVVDGTNFVVPTSHTLMSSPGFSAQLDSHF NPSHWD
LbCYP51B  361  QVVKETLRIHAPIHSIMRKVKQPLVVDGTNFVVPTSHTLMSSPGFSAQLDSHFVNPSHWD  420
                 Lm-αS  Lm-β-3-1  Lm-β-1-2  Lm-αT

                 Lm-αT  Lm-β-4-1  Lm-β-4-2  Lm-αU
LmCYP51B  420  PHRWDAGNSQYDEEADEKDDEKIDYGWGVVSKGTNSPYLPFGAGRHRCIGEQFAYLQLQT  479
               PHRWDAGNSQYDEEADEKDDEKIDYGWGVVSKGTNSPYLPFGAGRHRCIGEQFAYLQLQT
LbCYP51B  421  PHRWDAGNSQYDEEADEKDDEKIDYGWGVVSKGTNSPYLPFGAGRHRCIGEQFAYLQLQT  480
                 Lm-αU  Lm-β-4-1  Lm-β-4-2  Lm-αV

                 Lm-β-2-2  Lm-β-2-3
LmCYP51B  480  ILVAFVREFKLRNVGGSRDIIGTDYTSLFSRPLAPGIIEWERREKVE  526
               ILVAFVREFKLRNVGGS+DIIGTDYTSLFSRPLAPG++EWERREKVE
LbCYP51B  481  ILVAFVREFKLRNVGGSKDIIGTDYTSLFSRPLAPGVVEWERREKVE  527
                 Lm-β-2-2  Lm-β-2-3
```

SF 2

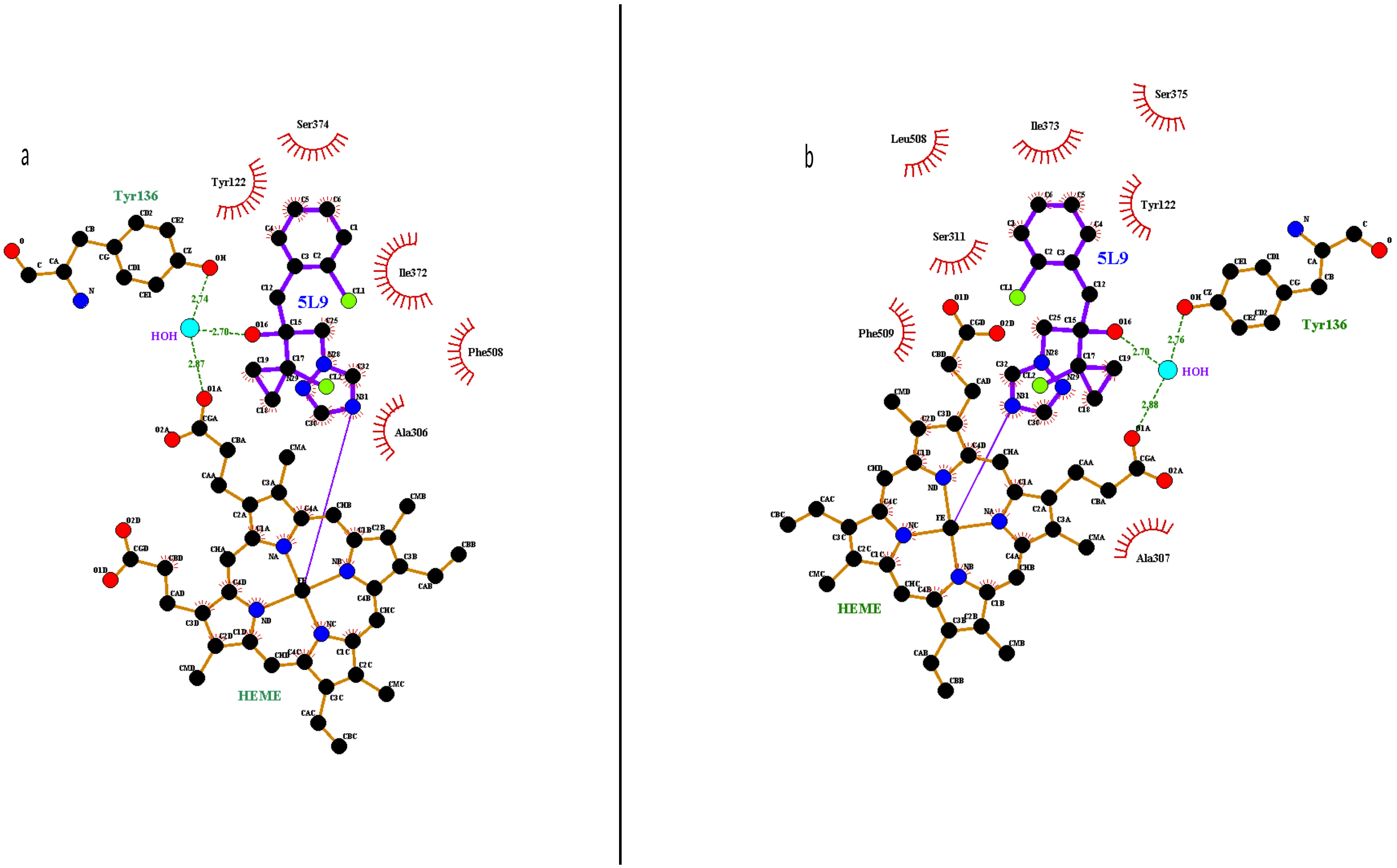
a
Tyr136
Tyr122
Ser374
5L9
Ile372
Phe508
HOH
2.74
2.70
2.87
Ala306
HEME
b
Leu508
Ile373
Ser375
Tyr122
Ser311
5L9
Phe509
Tyr136
HOH
2.76
2.70
2.88
Ala307
HEME

SF 3
